# Quantitative Scintigraphy Evaluated the Relationship between 131I Therapy and Salivary Glands Function in DTC Patients: A Retrospective Analysis

**DOI:** 10.1155/2022/7640405

**Published:** 2022-04-14

**Authors:** Xiaolan Lv, Liang Yin, Weiming Wu, Ning Li, Ruyi Zhang, Xue Li, Qiang Jia, Jian Tan, Peng Wang, Xiangqian Zheng, Xianghui He, Chao Huang, Dihua Li, Yan Wang, Zhaowei Meng

**Affiliations:** ^1^Department of Nuclear Medicine, Tianjin Medical University General Hospital, Tianjin 300052, China; ^2^Department of Ultrasound, Affiliated Hospital of Hebei University, Baoding 071000, China; ^3^Department of Nuclear Medicine, Pingjin Hospital, Characteristic Medical Center of Chinese People's Armed Police Forces, Tianjin 300162, China; ^4^Department of Thyroid and Neck Tumor, Tianjin Medical University Cancer Institute and Hospital, National Clinical Research Center for Cancer, Key Laboratory of Cancer Prevention and Therapy of Tianjin City, Tianjin 300060, China; ^**5**^ Department of General Surgery, Tianjin Medical University General Hospital, Tianjin 300052, China; ^6^Hull York Medical School, University of Hull, Hull, UK; ^7^Tianjin Key Laboratory of Acute Abdomen Disease Associated Organ Injury and ITCWM Repair, Institute of Acute Abdominal Diseases, Tianjin Nankai Hospital, Tianjin, China; ^8^Chinese Material Medical College, Tianjin State Key Laboratory of Modern Chinese Medicine, Tianjin University of Traditional Chinese Medicine, Tianjin 301617, China

## Abstract

**Purpose:**

Quantitative scintigraphy to evaluate salivary gland function changes in patients with differentiated thyroid cancer (DTC) after iodine-131 (^131^I) treatment.

**Methods:**

A total of 458 patients with DTC grouped by sex and age were included. Salivary gland scintigraphy was performed to evaluate salivary gland function before and after ^131^I treatment. The uptake fraction (UF), uptake index (UI), and excretion fraction (EF) of two pairs of parotid glands and submandibular glands were measured and compared. The Chi-square test was conducted according to function impairment count.

**Results:**

Salivary gland function in different age groups and sexes were quite different, especially for women <55 years old, who had decreased UF, UI, and EF of all four glands without basal injury. The secretion or uptake function of some salivary glands with basic function impairment before ^131^I treatment was increased after iodine treatment. Only a small percentage of males showed reduced functional parameters after several treatments. The most significant difference in the count of impairment for the four salivary glands were the first and third examinations, which was more evident in women. The submandibular gland had the most significant reduction in uptake.

**Conclusion:**

Changes in salivary gland function are more common in young females being treated for DTC. Impairment of salivary gland function is correlated with the number of treatments and the cumulative dose of ^131^I. Some salivary gland functions impaired before ^131^I treatment were enhanced in the early treatment.

## 1. Introduction

Differentiated thyroid cancer (DTC) is a common endocrine malignancy. According to statistics, 900,590 people were diagnosed with thyroid cancer in the United States. 52,070 people are expected to be diagnosed with thyroid cancer in 2019 [1]. Surgical treatment of thyroid cancer followed by removal of residual thyroid using iodine-131 (^131^I) is a common treatment approach, but ^131^I can induce salivary gland damage [2–4]. ^131^I is absorbed on the membranes of thyroid follicular cells and cancer cells through the reactive sodium iodide transporter (NIS) [5]. Salivary glands expressing NIS can also absorb ^131^I, and the accumulation of salivary gland ^131^I is about 30 to 40 times that of plasma levels [6]. The radiation dose of high-concentration ^131^I is sufficient to cause salivary gland damage and affect their function [7].

Salivary gland dysfunction is mainly reflected in decreased saliva secretion. Saliva is essential for the preservation of oral health. Saliva's functions include buffering, lubricating, mineralizing, and cleaning oral tissues [8]. Saliva also has antibacterial, antiviral, and antifungal properties [9]. Changes in the quantity or quality of saliva can affect the integrity of the oral tissues leading to the appearance of conditions like dental caries, periodontal diseases, and various other oral and pharyngeal disorders [10, 11]. In addition, salivary gland dysfunction is characterized by difficulty swallowing, dental disease, and loss of taste. DTC patients who had one or more ^131^I treatments may experience the above discomfort [12–14]. Their quality of life was affected. Therefore, determining and protecting salivary gland function in DTC patients should not be ignored. There are many examinations to assess salivary gland function, including salivary gland scintigraphy with ^99m^Tc-pertechnetate [3, 4, 15], neck ultrasonography [16], salivary flow rate measurement of the whole or individual gland [17]. ^99m^Tc-pertechnetate is commonly used in hospitals. Because it can quantify the uptake or secretory from individual salivary glands and calculate their function [18–20].

Through salivary gland scintigraphy, we found that some patients had impaired salivary gland function before ^131^I treatment. We defined it as an impairment of the basic function of the salivary glands. The Impairment of basic function could be associated with different factors, including Sjögren syndrome [21, 22], salivary gland obstructive disease [23], salivary gland infection [24], obesity and diabetes [25, 26], aging [27, 28] and so on. The changes in the salivary gland's function in these patients after ^131^I treatment are worth discussing.

This study aimed to analyze the changes in salivary gland uptake and excretion function following ^131^I treatment. And to study the relationships between the function change and different genders, age groups. The results provide clinical guidance for the protection of salivary function in DTC patients undergoing ^131^I treatment.

## 2. Materials and Methods

### 2.1. Patients

**Figure 1:**
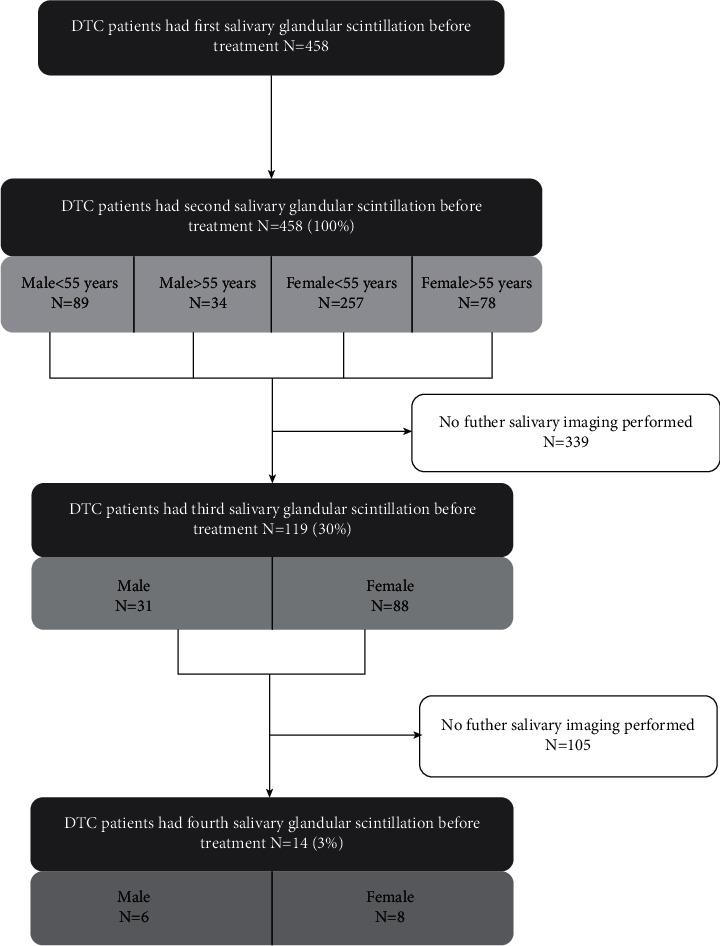
Case follow-up process. Salivary glandular scintillation imaging data were collected from DTC patients before the first, second, third, and fourth ^131^I treatments. The group was compared according to the number of patients in each treatment course.

A retrospective analysis of the hospital files from the Department of Nuclear Medicine of Tianjin Medical University General Hospital was in this study. The salivary gland scintigraphy parameters and inpatient treatment database of DTC patients from the hospital were used. We reviewed information for DTC patients who received ^131^I therapy from November 2014 to December 2018. All enrolled patients had two or more pre-hospital scintigraphy of salivary glands. A total of 458 patients with DTC grouped by sex and age were included. Patients with the above information missing were excluded. Total thyroidectomy was performed for all patients by thyroid surgeons, and DTC was diagnosed by postoperative pathology. According to the ATA Guidelines, we selected N1b or M1 DTC patients [2]. All patients received ^131^I treatment 6 weeks postoperatively. Before treatment with ^131^I, patients were advised to have a low-iodine diet for 3 weeks. After the first radioiodine treatment, patients in the study received one or more radioiodine treatments. Salivary gland function parameters were recorded by 370 MBq (10 mCi) ^99m^Tc-Pertechnetate salivary gland scintillation before ^131^I treatment. The interval between each treatment was ∼6 months. The protocol for evaluating salivary gland scintigraphy is shown in Figure 1. Patients with residual thyroid tissue were given 2.96 to 5.55 GBq (30–150 mCi) dosages for each treatment [2, 29].

### 2.2. Salivary Gland Imaging Protocol

According to our previous reports, pre-ablation salivary gland imaging was performed under thyroid-stimulating hormone (TSH) stimulation in the morning, 4 h before the first ^131^I intake [3, 4]. Patients were asked to fast before salivary gland imaging. Single-photon emission computed tomography was performed on a Discovery NM/CT 670 (General Electric Medical Systems, Chicago, IL, USA) while subjects laid on their back. A low-energy, parallel hole, high-resolution collimator was used with a peak value of 140 keV and a window width of 20%. Each patient received 370 MBq ^99m^Tc-pertechnetate intravenously through the cubital vein. After injection, the dynamic images were continuously shot on a 256 × 256 matrix at minute/frame with zoom 1.5 for 15 minutes. The patients were given 0.2 g oral vitamin C at the 8th minute after injection; they were instructed to chew quickly and then keep the tablet under the tongue for about 1 minute. To accurately calculate the delivered radioactivity dose, we measured the radioactivity count in the syringe before and after the injection. Patients underwent a radionuclide scan as described above before every ^131^I treatment. Salivary gland imaging was also performed under TSH stimulation.

### 2.3. Image Analysis

**Figure 2:**
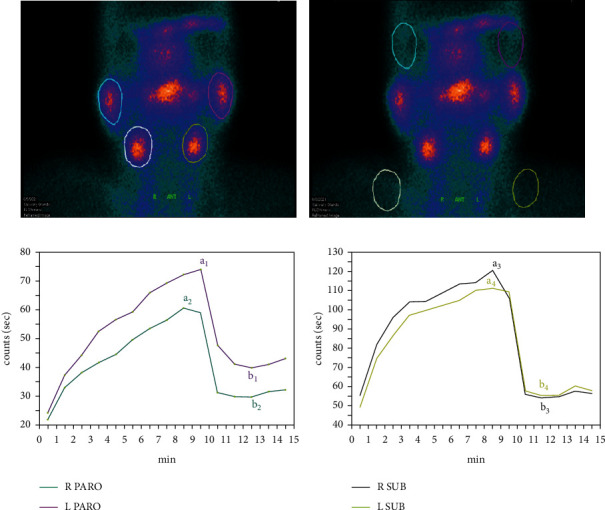
Physiological uptake and excretion of ^99m^Tc-pertechnetate in parotid and submandibular salivary glands detected by dynamic salivary gland scintigraphy. (a) A clear depiction of the ROIs and ROIs of the salivary and mandibular glands was obtained from all frames. (b) The parotid gland was in the bilateral temporal-orbital region, and the mandibular gland was in the bilateral supraclavicular region to draw similar background areas, and the background ROIs count was recorded. (c-d) Time-activity curve of each salivary gland. The ordinate of a_1-_a_4_ corresponds to the maximum minute count of each salivary gland. The ordinate of b1- b4 corresponds to the count of each salivary gland at the minimum uptake minute after vitamin C.

First, circular regions of interest (ROIs) were manually drawn on the parotid and submandibular glands. Parotid glands showed a similar unified background area in the bilateral temporal-orbital region, while submandibular glands appeared as a similar unified background area in the bilateral supraclavicular region. The sizes and positions of these ROIs remained the same for each scanning session. An imaging system was used to generate time-activity curves for ^99m^Tc-pertechnetate uptake and excretion in counts per minute. Based on these ROIs counts and the subsequent time-activity curves, the salivary gland functional indicators were derived using the following modified formulas [3, 4, 30, 31] (Figure 2):

#### 2.3.1. Uptake Fraction (UF)

UF =  (salivary gland maximum count minute–salivary gland background count corresponding to maximum count minute)/(count in the syringe before injection–count in the syringe after injection)

#### 2.3.2. Uptake Index (UI)

UI = (salivary gland maximum uptake count minute–salivary gland background count corresponding to maximum uptake count minute)/salivary gland background count corresponding to maximum uptake count minute

#### 2.3.3. Excretion Fraction (EF)

EF  = (salivary gland maximum uptake count minute–salivary gland minimum uptake count minute after vitamin C)/salivary gland background count corresponding to maximum uptake count minute

UI and UF reflect the uptake function of salivary glands, while EF reflects the secretion function.

### 2.4. Diagnostic Criteria for Salivary Gland Function

Salivary gland function impairment was established based on the diagnostic criteria of the Department of Nuclear Medicine, Tianjin Medical University General Hospital, with reference to previous studies and modified in our institute [18, 19, 32, 33]. Parameters obtained by salivary scintigraphy, the peak uptake (maximum salivary gland uptake count/second count at peak uptake) was set to <50 counts/s with reduced intake function, and EF was set to <30% with reduced secretory function. A reduced diagnosis of either or both of these above salivary glands is dysfunction. The patients with salivary gland dysfunction before the first admission were those with impaired basic salivary gland function.

### 2.5. Statistical Analysis

All data for males and females were analyzed separately and are presented as either mean ± standard deviation or median (upper quartile, lower quartiles). Statistical analysis was performed by using Statistical Package for Social Sciences (SPSS version 25.0, IBM Corp., Armonk, NY, USA) software. Mann-Whitney U tests were used to compare values of the same patient before and after the first treatment. Kruskal-Wallis tests were performed to evaluate and compare salivary gland function in patients who underwent multiple ^131^I treatments. After dividing men and women into separate groups, the distribution of salivary gland injuries before the first treatment and before the second to fourth treatments were analyzed by chi-square test.

## 3. Results

Among the 458 patients, most (72.9%) were female with a mean age of 46 ± 12 years (range 14–76 years). The lowest and highest doses of ^131^I were 30 and 550 mCi, respectively. Patient demographic data are summarized in Table 1.

From the scintigraphy examinations, the analysis according to age and sex groups evidenced salivary gland function is more sensitive in females than in males. The sensitivity of salivary gland function was ranked from large to small, in order of <55 years female, ≥55 years female, <55 years male, ≥55 years male (*p* = 0.05). After treatment with ^131^I, the UI, UF, or EF values of patients without impairment of basic salivary gland function tended to decrease. The secretion or uptake function of some salivary glands with basic function impairment before ^131^I treatment was increased after iodine treatment.  Table S1 summarizes the number of patients, age distribution, and cumulative dose before one or more ^131^I treatments. The UI, UF, and EF of each group were compared before and after ^131^I treatment (Tables 2–4).

We found that the difference between the first and third injury counts of salivary gland damage on salivary gland scintigraphy tests before hospitalization was the most significant. There were statistically significant differences between the four salivary glands in both the male and female groups. It was more pronounced in the female group (female, *P*=0.001; male, *P* < 0.05). The percentage of damaged salivary glands increased with the number of treatments in both sexes, while the percentage of normal salivary glands gradually decreased (Table 5, Figure 1S). The cumulative dose of ^131^I received by patients over several treatments is shown in Figure 2S. Chi-square test was performed to determine the relationship between the number of patients by sex and the impairment of salivary gland function before each ^131^I treatment (Table S2). There was a statistical difference in the left submandibular gland injury count between sexes before the first treatment (*p* < 0.05).

## 4. Discussion

Salivary gland damage is a common manifestation of DTC patients after ^131^I therapy [34]. ^131^I is mainly concentrated in the duct system of the salivary glands. The radiation causes debris buildup that narrows the lumen, and this obstruction can lead to an injurious process that results in glandular degeneration. Salivary gland scintigraphy examination is necessary for the objective evaluation of the reproducibility of salivary gland function [20]. These measurement parameters mainly assess gland uptake and secretion capacity [15, 35, 36]. We performed salivary gland scintigraphy to identify relevant salivary gland parameters and then analyzed the factors that affect salivary gland function changes in DTC patients after each ^131^I treatment.

### 4.1. Sex and Age

By comparing salivary glands without basic functional impairment, we found that functional changes were related to age and sex. The salivary glands of younger patients were more sensitive to ^131^I treatment than older patients, and female patients were more likely to show decreased function. Other studies have examined age- and sex-dependent differences in salivary gland function changes. Liu et al. assessed iodine dynamics and salivary gland dosimetry after ^131^I treatment and showed that women's parotid iodine intake was often higher than men's [37]. This suggests that female salivary glands are more susceptible to radiation, leading to decreased function following ^131^I treatment. Almeida et al. found that patient sex was associated with the uptake phase on salivary glands scintigraphy. Intake of all major salivary gland was decreased in men compared with women. Patient age was the strongest predictor of parotid gland dysfunction as it affects the stage of parotid gland uptake and elimination on salivary gland scintigraphy [38]. Another study revealed the presence of epidermal and nerve growth factors (EGF and NGF) in salivary glands and described their roles in cell growth and differentiation. They are detected at higher levels in the submandibular glands of males than females [39], indicating that male salivary gland cells have stronger repair and regeneration abilities. Animal studies showed sex-attributed differences in wound healing patterns in submandibular glands between male and female mice. In males, the number of convoluted tubules rich in EGF and NGF (involved in cell proliferation and neurogenesis, respectively) was higher than that in females [40, 41]. These sex- differences observed in mice may help explain why the incidence of salivary gland disease tends to be much higher in women than in men (i.e., approximately 9:1) [42].

### 4.2. Different Basic Functions

Before the first ^131^I treatment, we selected patients with basic impairment of salivary gland function. After the first ^131^I treatment, some salivary gland parameters were different in the female group, with most changes in the group younger than 55 years old. After the second ^131^I treatment, the bilateral submandibular gland EF values were different in females but not males. Interestingly, these altered functional parameters all showed an upward trend rather than the expected decline. We consider that this may be related to the compensatory increase in the function of glandular cells under certain stress states. Poradovskaia et al. showed that after ablating or removing one submandibular salivary gland in rats, the contralateral gland responded by increasing cell proliferation with concomitant increases in the size of the cells and nuclei by 10% and 17%, respectively. Burlage et al. observed that pilocarpine preconditioning induced proliferation of acinar and intercalated duct cells in rats, which could explain the observed enhanced compensatory response in salivary glands [43]. Compensatory proliferation is a mechanism to replace lost cells in rapidly cycling tissues [44]. After an initial singular dose ^131^I dose of 100 mCi, the salivary glands might increase uptake to maintain secretory function [4]. Our results are consistent with those of the above-mentioned studies. It is believed that salivary glands with slight damage in the basal state have a certain compensatory function. After the initial radiation injury, the compensatory function of gland cells is activated by stress, manifesting as increased uptake or excretion. With the increase of radiation dose and the passage of time after ^131^I treatment, this compensatory function gradually decreases or disappears. In this study, we also selected uninjured salivary glands and compared the functional parameters before and after ^131^I therapy. Notably, these salivary glands were more likely to be affected than those that were already impaired before treatment. Most showed functional reduction without the phenomenon of functional compensatory increase. This provides new ideas for clinical treatment. For example, salivary gland protection should be strengthened during ^131^I treatment, especially in patients with normal basal function.

### 4.3. Different Glands

The salivary gland imaging results after the first and second ^131^I treatments showed that the submandibular glands are more sensitive than the parotid ones, and the most common change was a decreased UF value representing impaired uptake. We analyzed whether this difference was related to salivary gland cell characteristics and salivary gland structure. Damage to the microvascular endothelial cells in salivary glands caused by radiotherapy is one of the causes of impaired gland function [45, 46]. It leads to microvascular dysfunction and the production of ceramide and reactive oxygen species (ROS) that can induce gland dysfunction. ROS scavengers are used to protect salivary gland function in radiotherapy patients [47]. By inhibiting or eliminating aberrant oxidation reactions, it is possible to reduce damage to salivary gland function caused by radiation. One study found that levels of salivary non-enzymatic antioxidants and antioxidant enzymes in the saliva secreted by the parotid gland were higher than those secreted by the submandibular salivary glands [48]. Therefore, salivary gland function changes due to parotid microvascular injury are not as serious as those caused by submandibular microvascular injury. Another group showed that the saliva-to-serum ^131^I concentration rates in the parotid gland of mice and humans were 0.59 and 4.6, espectively, while those in the submandibular gland were 5.1 and 6.9 [49]. The ^131^I concentration was higher in the submandibular glands of both species. An investigation showed that murine duct cells in the different salivary glands varied greatly in their ability to concentrate iodide, so it could be shown that ^131^I was mainly concentrated in the ducts of the submandibular glands in mice, with lower levels in the parotid gland and very little in sublingual gland ducts [50]. Because of their ability to concentrate ^131^I, the submandibular glands are more susceptible to radiation damage.

After the first ^131^I treatment, we performed a second scintigraphy scan. We found that the males younger than 55 and females older than 55 showed a tendency of decreased function of the left parotid gland compared to the right parotid gland. The differential changes in the left and right glands after radiation have been reported in several studies and may be due to the asymmetric concentration of radioactive iodine in the salivary glands [3, 15, 31].

### 4.4. Treatment Frequency and ^131^I Dose

The Chi-square test showed that the difference between the number of damaged and undamaged salivary glands in males and females increased significantly with the increase of treatment times, and the percentage of damaged glands also increased gradually. The number of treatments was correlated with the ^131^I cumulative dose. A correlation between radiation dose and salivary gland dysfunction was previously reported [51, 52]. A salivary glandular scintillation imaging study showed that ∼30% of salivary parenchymal function was lost following a single ^131^I dose of 6 GBq (162 mCi), with a cumulative dose of 35 GBq (945 mCi) resulting in complete loss of glandular function [53]. Parthasarathy and Crawford argued that significant side effects were rarely seen at doses <3.7 GBq (100 mCi) [54].

## 5. Limitations

Our results should be considered in the context of some limitations. First, this was a retrospective study with no survey to assess patient symptoms and signs, so it was not possible to add more conditions (e.g., dry mouth, difficulty swallowing, loss of taste) for case screening. Second, there was a small number of patients, especially among the group treated more than four times, which limited our analysis of salivary gland function in patients with DTC treated for more than 2–3 years. Third, clinical parameters and test data of some patients were missing. Finally, the lack of significant findings in males maybe because they only accounted for 27% of the study cohort.

## 6. Conclusions

This study quantitatively compared salivary scintigraphy parameters in DTC patients after multiple ^131^I treatments. Salivary gland function sensitivities are quite variable in different ages and sexes, with the highest sensitivity in women younger than 55. After treatment, the uptake or secretion function of some salivary glands with impaired basic function increased. Decreased salivary gland function is significantly related to the number of ^131^I treatments and the cumulative dose. The parotid glands have the most significant reduction in uptake.

## Figures and Tables

**Table 1:** Clinical characteristics of total patients.

Characteristic	Total	Male	Female	*P*
Age (years)	46 ± 12/14–76	46 ± 12/22–75	45 ± 12/14–76	0.442
Number/percentage		124/27%	334/73%	
^131^I activity (mCi)	128 ± 78/30–550	131 ± 68/30–450	126 ± 80/30–550	0.145

Age and ^131^I activity are stated as median ± standard deviation/range.

**Table 2:** Comparison of salivary gland function in patients before secondary treatmen.

Scintigraphic parameters	Male under 55 years						Male older than or equal to 55 years						Female under 55 years						Female older than or equal to 55 years					
	Damage before treatment	After firsttreatment	*P*	No damage before treatment	After firsttreatment	*P*	Damage before treatment	After firsttreatment	*P*	No damage before treatment	After firsttreatment	*P*	Damage before treatment	After firsttreatment	*P*	No damage before treatment	After firsttreatment	*P*	Damage before treatment	After firsttreatment	*P*	No damage before treatment	After firsttreatment	*P*
RP	Number:6			Number:83			Number:2			Number:32			Number:13			Number:244			Number:4			Number:74		
UF	0.039 (0.027–0.071)	0.057 (0.025–0.093)	0.173	0.043 (0.031–0.054)	0.039 (0.030–0.058)	0.806	0.016 (0.011–0.014)	0.018 (0.013–0.014)	0.180	0.047 (0.026–0.066)	0.043 (0.025–0.056)	0.100	0.023 (0.016–0.049)	0.043 (0.022–0.051)	0.116	0.043 (0.032–0.057)	0.038 (0.030–0.049)	0.000	0.025 (0.010–0.044)	0.027 (0.015–0.036)	0.715	0.047 (0.034–0.058)	0.037 (0.029–0.057)	0.218
UI	1.792 (1.337–2.293)	2.448 (1.117–4.080)	0.116	1.814 (1.425–2.377)	1.806 (1.424–2.423)	0.799	0.967 (0.589–0.862)	0.960 (0.707–0.732)	0.655	1.787 (1.176–2.175)	1.685 (0.974–1.949)	0.104	1.714 (1.221–2.363)	1.946 (1.364–2.643)	0.133	2.105 (1.621–2.618)	1.920 (1.467–2.369)	0.000	1.314 (0.774–2.224)	1.169 (0.836–1.466)	0.465	1.868 (1.514–2.604)	1.712 (1.349–2.410)	0.243
EF	0.109 (0.070–0.218)	0.501 (0.292–0.613)	0.046	0.473 (0.412–0.546)	0.461 (0.354–0.539)	0.064	0.306 (0.190–0.270)	0.268 (0.195–0.207)	0.655	0.444 (0.361–0.488)	0.396 (0.302–0.513)	0.161	0.316 (0.118–0.437)	0.465 (0.241–0.521)	0.055	0.496 (0.428–0.560)	0.467 (0.404–0.533)	0.001	0.176 (0.091–0.255)	0.359 (0.143–0.473)	0.144	0.508 (0.444–0.556)	0.475 (0.409–0.541)	0.113
LP	Number:3			Number:86			Number:2			Number:32			Number:17			Number:240			Number:8			Number:70		
UF	0.034 (0.019–0.048)	0.036 (0.016–0.040)	0.593	0.039 (0.030–0.050)	0.036 (0.027–0.050)	0.113	0.023 (0.011–0.023)	0.031 (0.012–0.034)	0.180	0.044 (0.034–0.072)	0.045 (0.025–0.067)	0.197	0.023 (0.013–0.036)	0.032 (0.020–0.048)	0.407	0.038 (0.029–0.053)	0.036 (0.028–0.048)	0.000	0.020 (0.011–0.042)	0.031 (0.023–0.047)	0.069	0.044 (0.030–0.061)	0.035 (0.024–0.047)	0.000
UI	0.805 (0.724–1.409)	1.197 (0.588–1.633)	0.285	1.874 (1.346–2.240)	1.689 (1.288–2.142)	0.079	1.072 (0.594–1.014)	1.312 (0.732–1.237)	0.180	1.858 (1.388–2.379)	1.567 (1.261–2.528)	0.443	1.465 (1.308–2.291)	1.770 (1.368–2.305)	0.227	2.013 (1.569–2.548)	1.841 (1.464–2.347)	0.000	1.464 (0.940–2.044)	1.560 (1.012–2.049)	0.401	2.050 (1.427–2.531)	1.630 (1.243–2.134)	0.005
EF	0.124 (0.104–0.154)	0.418 (0.132–0.443)	0.109	0.486 (0.427–0.544)	0.465 (0.377–0.549)	0.043	0.198 (0.053–0.244)	0.390 (0.227–0.358)	0.655	0.440 (0.359–0.511)	0.397 (0.304–0.498)	0.061	0.225 (0.120–0.357)	0.383 (0.339–0.467)	0.001	0.494 (0.420–0.564)	0.468 (0.394–0.535)	0.000	0.188 (0.167–0.240)	0.390 (0.261–0.487)	0.208	0.504 (0.432–0.588)	0.472 (0.366–0.535)	0.008
RS	Number:9			Number:80			Number:3			Number:31			Number:39			Number:218			Number:5			Number:73		
UF	0.033 (0.023–0.049)	0.024 (0.021–0.046)	0.767	0.058 (0.042–.0766)	0.048 (0.038–0.062)	0.000	0.023 (0.023–0.030)	0.036 (0.021–0.036)	0.285	0.057 (0.048–0.067)	0.045 (0.038–0.063)	0.011	0.034 (0.028–0.042)	0.032 (0.026–0.049)	0.944	0.047 (0.035–0.061)	0.042 (0.033–0.052)	0.000	0.010 (0.009–0.026)	0.019 (0.015–0.029)	0.345	0.062 (0.050–0.078)	0.055 (0.047–0.070)	0.006
UI	2.156 (1.363–2.733)	1.662 (1.297–2.530)	0.767	2.984 (2.540–3.957)	2.736 (2.209–3.705)	0.016	1.736 (0.886–1.989)	1.883 (1.282–2.093)	0.285	3.074 (2.399–3.531)	2.604 (2.127–3.239)	0.050	1.780 (1.358–2.158)	2.018 (1.582–2.324)	0.024	2.490 (2.002–3.307)	2.401 (1.997–2.945)	0.001	1.205 (.699–1.960)	1.402 (0.846–1.937)	0.893	3.034 (2.607–3.986)	2.923 (2.405–3.742)	0.008
EF	0.171 (0.157–0.190)	0.194 (0.175–0.254)	0.066	0.388 (0.313–0.470)	0.347 (0.256–0.444)	0.020	0.152 (0.038–0.281)	0.196 (0.050–0.358)	0.593	0.374 (0.304–0.421)	0.339 (0.238–0.400)	0.264	0.157 (0.116–0.181)	0.255 (0.180–0.341)	0.000	0.377 (0.285–0.457)	0.344 (0.264–0.413)	0.000	0.132 (0.072–0.150)	0.174 (0.090–0.257)	0.043	0.432 (0.338–0.520)	0.383 (0.290–0.488)	0.005
LS	Number:10			Number:79			One case			Number:31			Number:50			Number:207			Number:7			Number:71		
UF	0.027 (0.020–0.034)	0.025 (0.021–0.041)	0.241	0.052 (0.044–0.071)	0.045 (0.035–0.059)	0.000				0.061 (0.045–0.069)	0.048 (0.040–0.066)	0.010	0.029 (0.024–0.035)	0.029 (0.023–0.037)	0.490	0.045 (0.035–0.060)	0.042 (0.032–0.053)	0.000	0.017 (0.012–0.033)	0.022 (0.018–0.034)	0.398	0.061 (0.044–0.082)	0.050 (0.036–0.062)	0.000
UI	1.882 (1.247–2.158)	1.794 (1.499–2.296)	0.139	3.056 (2.488–4.031)	2.914 (2.371–3.532)	0.055				3.1515 (2.683–3.707)	2.973 (2.481–3.797)	0.348	1.917 (1.347–2.616)	2.015 (1.626–2.548)	0.420	2.877 (2.125–3.569)	2.630 (2.045–3.304)	0.004	1.974 (1.368–2.713)	2.239 (1.420–2.505)	0.735	3.120 (2.372–4.358)	2.809 (2.365–3.828)	0.003
EF	0.168 (0.149–0.187)	0.192 (0.134–0.251)	0.110	0.377 (0.282–0.460)	0.343 (0.236–0.425)	0.005				0.362 (0.290–0.432)	0.345 (0.252–0.423)	0.367	0.173 (0.139–0.198)	0.212 (0.171–0.291)	0.002	0.380 (0.306–0.475)	0.353 (0.277–0.428)	0.000	0.114 (0.063–0.174)	0.192 (0.142–0.336)	0.128	0.440 (0.324–0.499)	0.383 (0.275–0.462)	0.000

UF, UI, EF stated as median (upper quartile, lower quartiles), RP: Right parotid; LP: Left parotid; RS: Right submandibular; LS: Left submandibular; UF: Uptake fraction; UI: Uptake index; EF: Excretion fraction.

**Table 3:** Comparison of salivary gland function in patients before third treatment.

Scintigraphic parameters	Male group before third treatment								Female group before third treatment							
	Damage before treatment	After first treatment	After second treatment	*P*	No damage before treatment	After first treatment	After second treatment	*P*	Damage before treatment	After first treatment	After second treatment	*P*	No damage before treatment	After first treatment	After second treatment	*P*
RP	Number: 2				Number: 29				Number: 2				Number: 86			
UF	0.034 (0.018–0.033)	0.057 (0.019–0.067)	0.044 (0.012–0.055)	0.223	0.050 (0.031–0.068)	0.049 (0.032–0.060)	0.043 (0.026–0.061)	0.485	0.037 (0.010–0.045)	0.033 (0.010–0.039)	0.028 (0.008–0.034)	0.135	0.042 (0.031–0.055)	0.037 (0.028–0.048)	0.032 (0.021–0.041)	0.000
													III-II 0.005 III-I 0.000 ⋯ II-I 0.081			
UI	1.844 (1.054–1.712)	2.967 (0.865–3.586)	2.387 (0.864–2.717)	0.607	1.867 (1.527–2.391)	1.898 (1.440–2.819)	2.016 (1.277–3.038)	0.485	1.356 (0.673–1.360)	1.247 (0.533–1.336)	1.464 (0.588–1.607)	0.223	1.952 (1.549–2.479)	1.789 (1.460–2.188)	1.581 (1.137–2.102)	0.000
													III-II 0.014 III-I 0.000 II-I 0.098			
EF	0.254 (0.064–0.317)	0.506 (0.304–0.455)	0.299 (0.148–0.300)	0.607	0.486 (0.433–0.544)	0.478 (0.352–0.581)	0.493 (0.340–0.579)	0.422	0.194 (0.055–0.237)	0.418 (0.221–0.406)	0.412 (0.222–0.395)	1.000	0.485 (0.415–0.552)	0.4715 (0.419–0.539)	0.420 (0.246–0.521)	0.002
													III-II0.036 III-I 0.002 II-I 1.000			
LP	No case				Number: 31				Number: 5				Number: 83			
UF					0.041 (0.035–0.065)	0.045 (0.033–0.056)	0.036 (0.027–0.053)	0.206	0.019 (0.009–0.025)	0.025 (0.021–0.039)	0.021 (0.013–0.040)	0.165	0.040 (0.029–0.051)	0.034 (0.027–0.045)	0.032 (0.021–0.042)	0.000
													III-II 0.309 III-I 0.000 II-I 0.016			
UI					1.971 (1.523–2.460)	1.897 (1.348–2.457)	1.615 (1.359–2.576)	0.597	0.861 (0.687–1.776)	1.164 (1.010–1.879)	1.332 (.916–2.084)	0.247	2.006 (1.393–2.281)	1.717 (1.319–2.143)	1.686 (1.231–2.194)	0.008
													III-II 1.000 III-I 0.020 II-I 0.025			
EF					0.510 (0.442–0.558)	0.464 (0.402–0.550)	0.455 (0.347–0.545)	0.086	0.166 (0.102–0.382)	0.368 (0.292–0.439)	0.328 (0.173–0.458)	0.247	0.479 (0.420–0.544)	0.472 (0.394–0.523)	0.423 (0.326–0.514)	0.010
													III-II 0.392 III-I 0.008 II-I 0.392			
RS	Number: 2				Number: 29				Number: 6				Number: 82			
UF	0.034 (0.017–0.033)	0.033 (0.016–0.034)	0.032 (0.021–0.027)	1.000	0.058 (0.046–0.080)	0.054 (0.042–0.066)	0.051 (0.038–0.065)	0.018	0.050 (0.033–0.064)	0.044 (0.032–0.068)	0.044 (0.018–0.074)	0.607	0.052 (0.039–0.068)	0.048 (0.035–0.057)	0.043 (0.034–0.054)	0.000
					III-II 1.000 III-I 0.017 II-I 0.147								III-II 0.823 III-I 0.000 II-I 0.004			
UI	1.553 (0.664–1.665)	1.821 (0.961–1.770)	1.636 (1.189–1.265)	0.607	3.171 (2.438–3.936)	3.105 (2.284–3.663)	3.052 (2.415–3.624)	0.639	1.650 (1.266–2.771)	2.251 (2.099–3.090)	2.024 (1.318–3.273)	0.115	2.685 (2.161–3.528)	2.715 (2.021–3.318)	2.395 (2.010–3.092)	0.011
													III-II 0.930 III-I 0.009 II-I 0.153			
EF	0.096 (0.029–0.115)	0.122 (0.038–0.146)	0.073 (0.032–0.078)	0.223	0.381 (0.335–0.467)	0.375 (0.271–0.416)	0.396 (0.318–0.468)	0.343	0.159 (0.122–0.169)	0.339 (0.248–0.382)	0.343 (0.191–0.439)	0.042	0.404 (0.308–0.498)	0.367 (0.291–0.435)	0.332 (0.242–0.430)	0.000
									III-II 0.773 III-I 0.021 II-I 0.043 ^b^				III-II 0.414 III-I0 .000 II-I0 .024			
LS	Number: 2				Number: 29				Number: 9				Number: 79			
UF	0.031 (0.011–0.036)	0.033 (0.009–0.040)	0.023 (0.009–0.025)	0.607	0.056 (0.044–0.079)	0.048 (0.040–0.065)	0.050 (0.040–0.059)	0.024	0.033 (0.023–0.051)	0.034 (0.020–0.040)	0.030 (0.021–0.043)	0.895	0.050 (0.039–0.068)	0.045 (0.034–0.055)	0.041 (0.033–0.054)	0.000
					III-II 1.000 III-I 0.018 II-I 0.018								III-II0.529 III-I 0.000 II-I 0.016			
UI	1.482 (0.656–1.568)	1.570 (0.645–1.711)	1.030 (0.421–1.125)	0.223	3.584 (2.667–4.089)	3.236 (2.655–3.797)	3.053 (2.546–3.685)	0.166	1.891 (1.437–2.864)	2.019 (1.591–2.928)	1.932 (1.408–2.687)	0.641	2.979 (2.219–3.580)	2.687 (2.140–3.362)	2.542 (1.977–3.191)	0.003
													III-II 1.000 III-I 0.003 II-I .051			
EF	0.169 (0.123–0.131)	0.072 (0.000–0.108)	0.034 (0.016–0.034)	0.223	0.418 (0.316–0.492)	0.372 (0.295–0.436)	0.404 (0.321–0.528)	0.166	0.169 (0.119–0.199)	0.336 (0.229–0.386)	0.235 (0.165–0.386)	0.050	0.395 (0.300–0.499)	0.365 (0.273–0.444)	0.323 (0.233–0.435)	0.000

UF, UI, EF stated as median (upper quartile, lower quartiles). Examination before first, second and third treatments expressed as I, II, and III. The values were *p* values after pairwise comparison of I, II, III. RP: Right parotid; LP: Left parotid; RS: Right submandibular; LS: Left submandibular; UF: Uptake fraction; UI: Uptake index; EF: Excretion fraction.

**Table 4:** Comparison of salivary gland function in patients before fourth treatment.

Scintigraphy parameters	Male group before fourth treatment number: 6					Female group before fourth treatment number: 8				
	Before treatment	After first treatment	After second treatment	After third treatment	*P*	Before treatment	After first treatment	After second treatment	After third treatment	*P*
RP										
UF	0.054 (0.024–0.072)	0.072 (0.035–0.089)	0.051 (0.025–0.077)	0.052 (0.015–0.076)	0.896	0.047 (0.043–0.056)	0.032 (0.031–0.046)	0.033 (0.018–0.037)	0.010 (0.009–0.042)	0.013
						VI-III 1.000 VI-II.231 VI-I .011 III-II 1.000 III-I .231 II-1 1.000				

UI	2.237 (1.492–2.725）	2.3137 (1.691–3.522）	2.252 (1.425–3.257）	2.127 (1.323–2.668）	0.706	2.371 (1.860–2.885)	1.690 (1.537–2.151)	1.388 (0.808–1.844)	0.707 (0.529–1.887)	0.003
						VI-III 1.000 VI-II0.317 VI-I .003 III-II 1.000 III-I 0.040 II-1 0.728				

EF	0.485 (0.330–0.559）	0.542 (0.460–0.705）	0.458 (0.364–0.547）	0.403 (0.004–0.548）	0.204	0.543 (0.500–0.567）	0.478 (0.439–0.487）	0.158 (0.023–0.446）	0.050 (0.022–0.407）	0.001
						VI-III 1.000 VI-II0.071 VI-I .006 III-II 0.199 III-I 0.022 II-1 1.000				

LP										
UF	0.040 (0.035–0.065）	0.052 (0.039–0.067)	0.041 (0.029–0.070)	0.031 (0.010–0.045)	0.849	0.041 (0.035–0.050）	0.035 (0.027–0.048)	0.033 (0.026–0.044)	0.025 (0.018–.035)	0.034
						VI-III 0.137 VI-II 0.231 VI-I 0.043 III-II 1.000 III-I 1.000 II-1 1.000				

UI	1.958 (1.661–2.357)	2.3628 (1.468–2.616)	1.623 (1.307–3.080)	1.675 (1.213–1.995)	0.978	2.051 (1.765–2.396)	1.684 (1.534–2.175)	1.635 (0.963–2.104)	1.576 (0.472–2.029)	0.010
						VI-III 1.000 VI-II 1.000 VI-I .040 III-II 1.000 III-I 0.012 II-I0.317				

EF	0.515 (0.480–0.589)	0.487 (0.385–0.575)	0.419 (0.216–0.548)	0.287 (0.047–0.48)	0.038	0.475 (0.454–0.526)	0.445 (0.297–0.485)	0.411 (0.031–0.501)	0.429 (0.016–0.518)	0.522
	I-III 1.000 VI-II 1.000 VI-I 0.044 III-II 1.000 III-1 0.152 II-1 1.000									

RS										
UF	0.053 (0.040–0.064)	0.057 (0.036–0.076)	0.050 (0.030–0.067)	0.040 (0.014–0.076)	0.284	0.052 (0.041–0.059)	0.039 (0.035–0.048)	0.043 (0.035–0.054)	0.037 (0.030–0.044)	0.021
						VI-III .043 VI-II .586 VI-I .043 III-II 1.000 III-I 1.000 II-1 1.000				

UI	2.147 (1.884–2.778)	3.041 (2.015–3.985)	2.703 (1.927–3.185)	2.726 (1.660–4.742)	0.572	2.988 (2.479–3.495)	2.465 (2.125–3.067)	2.649 (2.306–2.857)	2.447 (2.239–2.601)	0.119
EF	0.386 (0.338–0.444)	0.352 (0.246–0.471)	0.394 (0.315–0.466)	0.422 (0.181–0.475)	0.940	0.437 (0.373–0.455)	0.282 (0.254–0.352)	0.284 (0.218–0.405)	0.348 (0.143–0.437)	0.010
						VI-III 1.000 VI-II 1.000 VI-I 0.317 III-II 1.000 III-I0.040 II-1 0.012				

LS										
UF	0.052 (0.044–0.056)	0.045 (0.043–0.078)	0.054 (0.041–0.063)	0.045 (0.015–0.070)	0.849	0.049 (0.042–0.056)	0.042 (0.030–0.047)	0.042 (0.034–0.049)	0.037 (0.030–0.040)	0.392
UI	2.605 (1.676–3.068)	3.420 (2.807–3.783)	3.023 (2.450–3.487)	2.904 (2.145–3.613)	0.706	2.550 (2.338–3.107)	2.620 (1.897–3.204)	2.666 (2.135–3.045)	2.472 (2.344–2.949)	0.789
EF	0.397 (0.350–0.456)	0.371 (0.255–0.427)	0.420 (0.297–0.504)	0.376 (0.166–0.453)	0.392	0.332 (0.269–0.407)	0.289 (0.259–0.355)	0.274 (0.194–0.405)	0.327 (0.129–0.415)	0.327

UF, UI, EF stated as median (upper quartile, lower quartiles). Examination before first, second, and third treatments expressed as I, II, III, and IV. The values were *P* values after pairwise comparison of I, II, III, IV. RP: Right parotid; LP: Left parotid; RS: Right submandibular; LS: Left submandibular; UF: Uptake fraction; UI: Uptake index; EF: Excretion fraction.

**Table 5:** The number of salivary gland injuries before the last three treatments compared with the first treatment.

Scintigraphic parameters	Male										Female								
	Damage	First	Second	*P*	First	Third	*P*	First	Fourth	*P*	First	Second	*P*	First	Third	*P*	First	Fourth	*P*
RP	Hurt	8 (6.5%)	8 (6.5%)	1.000	8 (6.5%)	8 (25.8%)	0.005	8 (6.5%)	2 (33.3%)	0.069	17 (5.1%)	16 (5.1%)	0.859	17 (5.1%)	29 (33%)	0.001	17 (5.1%)	5 (62.5%)	0.001
	No hurt	115 (93.5%)	115 (93.5%)		115 (93.5%)	23 (74.2%)		115 (93.5%)	4 (66.7%)		318 (94.9%)	319 (94.9%)		318 (94.9%)	59 (67%)		318 (94.9%)	3 (37.5%)	

LP	Hurt	5 (4.1%)	9 (7.3%)	0.271	5 (4.1%)	7 (22.6%)	0.003	5 (4.1%)	3 (50%)	0.003	25 (7.5%)	23 (6.9%)	0.881	25 (7.5%)	24 (27.3%)	0.001	25 (7.5%)	2 (25%)	0.125
	No hurt	118 (95.9%)	114 (92.7%)		118 (95.9%)	24 (87.4%)		118 (95.9%)	3 (50%)		310 (92.5%)	312 (93.1%)		310 (92.5%)	64 (72.7%)		310 (92.5%)	6 (75%)	

RS	Hurt	12 (9.8%)	19 (15.4%)	0.179	12 (9.%)	8 (25.8%)	0.032	12 (9.8%)	2 (33.3%)	0.128	44 (13.1%)	48 (14.3%)	0.653	44 (13.1%)	36 (40.9%)	0.001	44 (13.1%)	2 (25%)	0.292
	No hurt	111 (90.2%)	104 (84.6%)		111 (90.2%)	23 (74.2%)		111 (90.2%)	4 (66.7%)		291 (86.9%)	287 (85.7%)		291 (86.9%)	52 (59.1%)		291 (86.9%)	6 (75%)	

LS	Hurt	11 (8.9%)	22 (17.9%)	0.040	11 (8.9%)	8 (25.8%)	0.027	11 (8.9%)	2 (33.3%)	0.112	57 (17%)	62 (18.5%)	0.613	57 (17%)	39 (44.3%)	0.001	57 (17%)	4 (50%)	0.036
	No hurt	112 (91.1%)	101 (82.1%)		112 (91.1%)	23 (74.2%)		112 (91.1%)	4 (66.7%)		278 (83%)	273 (81.5%)		278 (83%)	49 (55.7%)		278 (83%)	4 (50%)	

The sequence number of examinations before treatments. Expressed as number of cases (values of number of cases/total cases of this examination before treatments ×  100%). RP: Right parotid; LP: Left parotid; RS: Right submandibular; LS: Left submandibular.

## Data Availability

The data generated in the study are included in this article. The database is available upon request.

## Supplementary Materials

Supplementary Materials Table S1 summarizes the number of patients, age distribution, and cumulative dose before one or more ^131^I treatments. Table S2: Chi-square test was performed to determine the relationship between the number of patients by sex and the impairment of salivary gland function before each ^131^I treatment. There was a statistical difference in the left submandibular gland injury count between sexes before the first treatment (*p* < 0.05). Figure S1: The percentage of damaged salivary glands increased with the number of treatments in both sexes. Figure S2: The cumulative dose of ^131^I received by patients over several treatments.
