# Supplementary material for: Quantitative Scintigraphy Evaluated the Relationship between 131I Therapy and Salivary Glands Function in DTC Patients: A Retrospective Analysis
Source: J Healthc Eng. 2022 Apr 14;2022:7640405. doi: 10.1155/2022/7640405 (PMC9023193; doi:10.1155/2022/7640405)
Supplement: Supplementary Materials — Table S1 summarizes the number of patients, age distribution, and cumulative dose before one or more 131I treatments. Table S2: Chi-square test was performed to determine the relationship between the number of patients by sex and the impairment of salivary gland function before each 131I treatment. There was a statistical difference in the left submandibular gland injury count between sexes before the first treatment (p < 0.05). Figure S1: The percentage of damaged salivary glands increased with the number of treatments in both sexes. Figure S2: The cumulative dose of 131I received by patients over several treatments. [file 7640405.f1.zip › 7640405.f1/Table S1 (1).docx]

Table S1 Clinical characteristics of patients before each treatment

|  |  | First treatment | | | | |  | Second treatment | | | |  | Third treatment | | | |
| --- | --- | --- | --- | --- | --- | --- | --- | --- | --- | --- | --- | --- | --- | --- | --- | --- |
|  | Total | Male <55 | Male  ≥55 | Female <55 | Female≥55 | P |  | Total | Male | Female | P |  | Total | Male | Female | P |
| Age（years） | 46±12 / 14-76 | 40±8/  22- 54 | 62±5/  55-75 | 41±9/  14-54 | 60±5/ 55-76 | 0.000 |  | 46±12 / 18-72 | 48±12/  27-72 | 46±11/  18-66 | 0.311 |  | 40±11 **/** 26-63 | 46±13/  26-48 | 35±8/  18-66 | 0.098 |
| Number | 458 | 89 | 34 | 257 | 78 | 0.213 |  | 119 | 31 | 88 | 0.157 |  | 14 | 6 | 8 | 0.157 |
| ^131^I activity (mCi) | 128±78/30 -200 | 102±2/30-150 | 107±27/ 30-200 | 96±30 / 30-200 | 96±27/30-150 | 0.552 |  | 210±48/80 -350 | 202±41/80-300 | 212± 51/  80-350 | 0.327 |  | 365±96/  260 -550 | 340±56/300-450 | 390±125/260-550 | 0.403 |

Age and ^131^I activity are stated as median ± standard deviation/ range.
