# Supplementary material for: Quantitative Scintigraphy Evaluated the Relationship between 131I Therapy and Salivary Glands Function in DTC Patients: A Retrospective Analysis
Source: J Healthc Eng. 2022 Apr 14;2022:7640405. doi: 10.1155/2022/7640405 (PMC9023193; doi:10.1155/2022/7640405)
Supplement: Supplementary Materials — Table S1 summarizes the number of patients, age distribution, and cumulative dose before one or more 131I treatments. Table S2: Chi-square test was performed to determine the relationship between the number of patients by sex and the impairment of salivary gland function before each 131I treatment. There was a statistical difference in the left submandibular gland injury count between sexes before the first treatment (p < 0.05). Figure S1: The percentage of damaged salivary glands increased with the number of treatments in both sexes. Figure S2: The cumulative dose of 131I received by patients over several treatments. [file 7640405.f1.zip › 7640405.f1/Table S2 (1).docx]

Table S2 Relationship between sex and functional impairment of salivary glands before each treatment

|  | Before first treatment | | | |  | Before second treatment | | | |  | Before third treatment | | | |  | Before fourth treatment | | | |
| --- | --- | --- | --- | --- | --- | --- | --- | --- | --- | --- | --- | --- | --- | --- | --- | --- | --- | --- | --- |
|  | Damage | F | M | P |  | Damage | F | M | P |  | Damage | F | M | P |  | Damage | F | M | P |
| RP | Hurt | 8 | 17 | 0.551 |  | Hurt | 8 | 16 | 0.462 |  | Hurt | 6 | 21 | 0.541 |  | Hurt | 2 | 6 | 0.315 |
|  | No hurt | 115 | 318 |  |  | No hurt | 115 | 319 |  |  | No hurt | 27 | 69 |  |  | No hurt | 4 | 3 |  |
|  |  |  |  |  |  |  |  |  |  |  |  |  |  |  |  |  |  |  |  |
| LP | Hurt | 5 | 25 | 0.193 |  | Hurt | 9 | 23 | 0.867 |  | Hurt | 5 | 19 | 0.460 |  | Hurt | 2 | 3 | 1.000 |
|  | No hurt | 118 | 310 |  |  | No hurt | 114 | 312 |  |  | No hurt | 28 | 71 |  |  | No hurt | 4 | 6 |  |
|  |  |  |  |  |  |  |  |  |  |  |  |  |  |  |  |  |  |  |  |
| RS | Hurt | 12 | 44 | 0.328 |  | Hurt | 19 | 48 | 0.764 |  | Hurt | 3 | 14 | 0.361 |  | Hurt | 2 | 3 | 1.000 |
|  | No hurt | 111 | 291 |  |  | No hurt | 104 | 287 |  |  | No hurt | 31 | 79 |  |  | No hurt | 4 | 6 |  |
|  |  |  |  |  |  |  |  |  |  |  |  |  |  |  |  |  |  |  |  |
| LS | Hurt | 11 | 57 | 0.031 |  | Hurt | 22 | 62 | 0.879 |  | Hurt | 4 | 19 | 0.257 |  | Hurt | 2 | 3 | 1.000 |
|  | No hurt | 112 | 278 |  |  | No hurt | 101 | 273 |  |  | No hurt | 29 | 71 |  |  | No hurt | 4 | 6 |  |

RP: Right parotid; LP: Left parotid; RS: Right submandibular; LS: Left submandibular, F: Female number; M: Male number.
