# Supplementary figures and images for: Quantitative Scintigraphy Evaluated the Relationship between 131I Therapy and Salivary Glands Function in DTC Patients: A Retrospective Analysis
Source: J Healthc Eng. 2022 Apr 14;2022:7640405. doi: 10.1155/2022/7640405 (PMC9023193; doi:10.1155/2022/7640405)

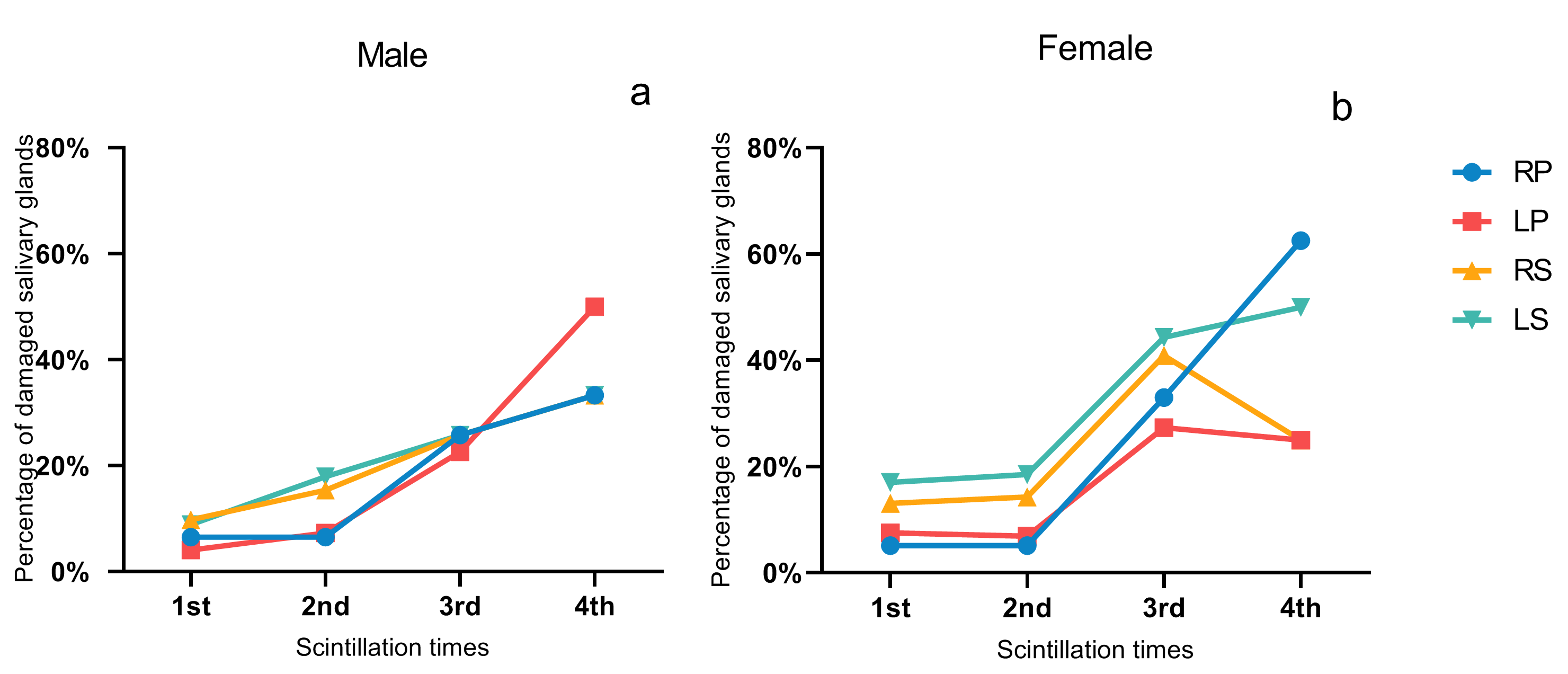

Supplement: Supplementary Materials — Table S1 summarizes the number of patients, age distribution, and cumulative dose before one or more 131I treatments. Table S2: Chi-square test was performed to determine the relationship between the number of patients by sex and the impairment of salivary gland function before each 131I treatment. There was a statistical difference in the left submandibular gland injury count between sexes before the first treatment (p < 0.05). Figure S1: The percentage of damaged salivary glands increased with the number of treatments in both sexes. Figure S2: The cumulative dose of 131I received by patients over several treatments. [file 7640405.f1.zip › 7640405.f1/Figure S1 (1).docx]

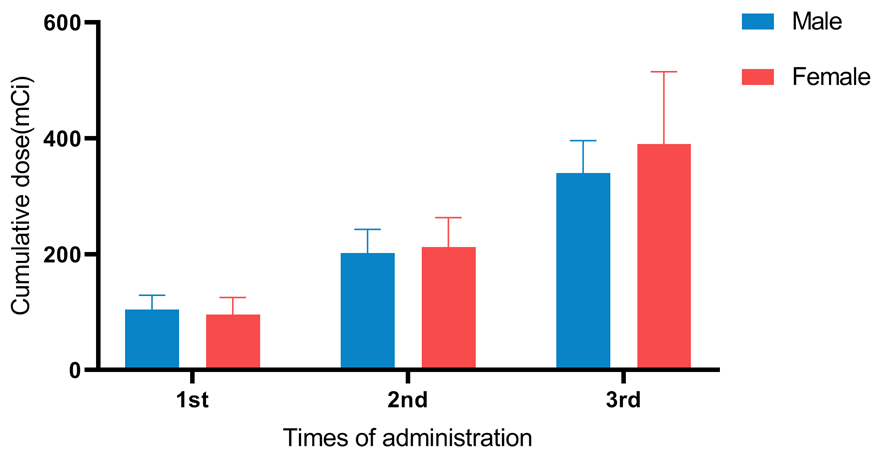

Supplement: Supplementary Materials — Table S1 summarizes the number of patients, age distribution, and cumulative dose before one or more 131I treatments. Table S2: Chi-square test was performed to determine the relationship between the number of patients by sex and the impairment of salivary gland function before each 131I treatment. There was a statistical difference in the left submandibular gland injury count between sexes before the first treatment (p < 0.05). Figure S1: The percentage of damaged salivary glands increased with the number of treatments in both sexes. Figure S2: The cumulative dose of 131I received by patients over several treatments. [file 7640405.f1.zip › 7640405.f1/Figure S2 (1).docx]
